# Trachoma, Anti-Pgp3 Serology, and Ocular *Chlamydia trachomatis* Infection in Papua New Guinea

**DOI:** 10.1093/cid/ciaa042

**Published:** 2020-01-22

**Authors:** Colin K Macleod, Robert Butcher, Sarah Javati, Sarah Gwyn, Marinjho Jonduo, Mohammad Yazid Abdad, Chrissy H Roberts, Drew Keys, Samuel Peter Koim, Robert Ko, Jambi Garap, David Pahau, Wendy Houinei, Diana L Martin, William S Pomat, Anthony W Solomon

**Affiliations:** 1 London School of Hygiene and Tropical Medicine, London, United Kingdom; 2 Papua New Guinea Institute of Medical Research, Goroka, Papua New Guinea; 3 Centers for Disease Control and Prevention, Atlanta, Georgia, USA; 4 National Centre for Infectious Diseases, Singapore; 5 Brien Holden Vision Institute Foundation, Sydney, Australia; 6 PNG Eye Care, Port Moresby, Papua New Guinea; 7 Department of Ophthalmology, Port Moresby General Hospital, Port Moresby, Papua New Guinea; 8 Department of Ophthalmology, Boram General Hospital, Wewak, Papua New Guinea; 9 Neglected Tropical Diseases, National Department of Health, Port Moresby, Papua New Guinea

**Keywords:** trachoma, ocular *Chlamydia trachomatis*, anti-Pgp3 antibodies, Papua New Guinea, neglected tropical diseases

## Abstract

**Background:**

In Melanesia, the prevalence of trachomatous inflammation–follicular (TF) suggests that public health–level interventions against active trachoma are needed. However, the prevalence of trachomatous trichiasis is below the threshold for elimination as a public health problem and evidence of conjunctival infection with trachoma’s causative organism (*Chlamydia trachomatis* [CT]) is rare. Here, we examine the prevalence of ocular infection with CT and previous exposure to CT in three evaluation units (EUs) of Papua New Guinea.

**Methods:**

All individuals aged 1–9 years who were examined for clinical signs of trachoma in 3 Global Trachoma Mapping Project EUs were eligible to take part in this study (N = 3181). Conjunctival swabs were collected from 349 children with TF and tested by polymerase chain reaction to assess for ocular CT infection. Dried blood spots were collected from 2572 children and tested for anti-Pgp3 antibodies using a multiplex assay.

**Results:**

The proportion of children with TF who had CT infection was low across all 3 EUs (overall 2%). Anti-Pgp3 seroprevalence was 5.2% overall and there was no association between anti-Pgp3 antibody level and presence of TF. In 2 EUs, age-specific seroprevalence did not increase significantly with increasing age in the 1- to 9-year-old population. In the third EU, there was a statistically significant change with age but the overall seroprevalence and peak age-specific seroprevalence was very low.

**Conclusions:**

Based on these results, together with similar findings from the Solomon Islands and Vanuatu, the use of TF to guide antibiotic mass drug administration decisions in Melanesia should be reviewed.

## Teaser

In Papua New Guinea (PNG), prevalence of trachomatous trichiasis is very low. This study demonstrates that prevalence and transmission of ocular *Chlamydia trachomatis*, a driver of progressive trachomatous disease, is much lower in PNG than in other settings with similar active trachoma prevalence.

In the 1950s, a large eye health survey suggested that trachoma was the second most common cause of binocular blindness in Papua New Guinea (PNG) [1]. However, more recent observers have noted trachoma in PNG to be mild in presentation, on the basis that many children have clinical signs of active (inflammatory) disease whereas very few adults have trachomatous trichiasis (TT) [2, 3]. A recent nationwide rapid assessment of avoidable blindness only identified 1 case of corneal opacity attributed to trachoma [4]. In October–December 2015, trachoma was mapped in 6 evaluation units (EUs) in PNG as part of the Global Trachoma Mapping Project [5]. The trachomatous inflammation–follicular (TF) prevalence in children aged 1–9 years in those EUs ranged from 6.0% to 12.2%, exceeding the World Health Organization (WHO) threshold for intervention (TF ≥ 5%) in every EU. In 4 EUs, the TF prevalence was  > 10%, for which 3 rounds of antibiotic mass drug administration (MDA) are recommended before resurvey. However, the TT prevalence in those aged ≥ 15 years in all EUs was below the WHO threshold above which TT is considered a public health problem (0.2%). In fact, no cases of TT were found in 5 of 6 EUs [6].

Population-based mapping from neighboring Pacific Island countries Fiji, Solomon Islands, and Vanuatu suggests that, as in PNG, TF is common in children whereas the prevalence of TT in adults is almost universally below 0.2% [7–10]. In the Solomon Islands and Vanuatu, nucleic acid amplification–based testing for ocular *Chlamydia trachomatis* (CT) infection has demonstrated that the prevalence of infection is much lower than in districts outside of Melanesia with similar TF prevalence [11]. This finding is complemented by serological data suggesting that ocular CT transmission is low [12]. These data suggest that TF in Melanesian countries may not be entirely due to CT. Some have hypothesized that estimates of the prevalence of active trachoma are inflated by follicular conjunctivitis of an as-yet unidentified etiology. Alternatively, the association between TF and infection may be weakened by temporal change (for example, an ongoing decrease in the prevalence of trachoma throughout the region leading to less frequent exposure to CT, arrested immunity, and prolonged clinical inflammation). Understanding the etiology of follicular conjunctivitis in these settings is critical for directing the use of appropriate prevention and treatment measures [13, 14].

This pattern of ocular CT infection in other countries of the Western Pacific region caused policy makers in PNG to question whether intervention with the A, F, and E components of the SAFE strategy (surgery, antibiotics, facial cleanliness and environmental improvement) would be required for local trachoma control [15]. The aim of this study was to supplement routine trachoma mapping with CT infection data to help guide local policy development and expand the evidence base on trachoma in Melanesia.

## MATERIALS AND METHODS

### Ethical Considerations

Ethical approval for the underlying Global Trachoma Mapping Project survey was granted by the PNG Institute of Medical Research (PNG-IMR) Institutional Review Board (1606), the PNG Medical Research Advisory Committee (16.36 and 15.20), and the London School of Hygiene and Tropical Medicine (LSHTM) Observational Ethics Committee (6319 and 8355). Ethics approval for collection and testing of samples was granted by the PNG Medical Research Advisory Committee (16.36). Quality control activities related to PCR were approved by LSHTM (11950). Centers for Disease Control and Prevention (CDC) investigators were considered to be nonengaged with human subjects. A parent or guardian gave written informed consent for each child to take part in this study.

### Survey Design

**Figure 1.**
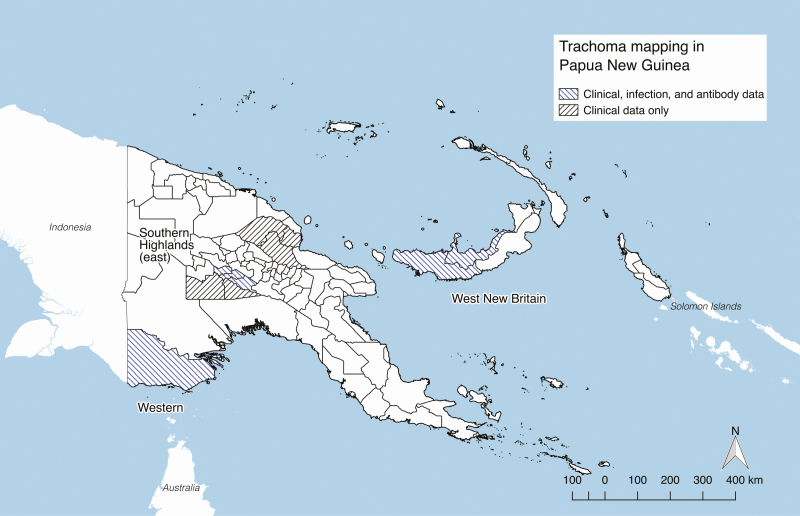
Districts of Papua New Guinea where trachoma mapping took place in October–December 2015. Provinces where specimens were collected for serology and ocular *Chlamydia trachomatis* infection testing are named.

The survey design, examination methodology, and quality control measures are described in detail elsewhere [6, 16, 17]. Three EUs were selected in advance for specimen collection based on the expectation of national healthcare staff that TF would be more common in those areas. The selected EUs are shown in Figure 1. In each EU, using sample size calculations based on an expected TF prevalence of 10% in children aged 1–9 years, 27 villages were selected. In each village, in October–December 2015, 30 households were randomly selected by listing all village households in collaboration with the village head and drawing lots. Ocular swabs were collected from children aged 1–9 years who had TF in 1 or both eyes and dried blood spot (DBS) samples were collected from all examined children aged 1–9 years. This age group was selected because they are considered to be at highest risk of infection and active trachoma [18].

### Ocular CT Infection

Conjunctival swabs were collected from children with TF using previously described methods [12]. As usual in studies of ocular CT infection [19–22], to contain study costs, a maximum of 1 eye was swabbed per person; where an individual’s eye was swabbed, we swabbed the right eye. The swab head was immediately placed into a polyethylene tube and stored in a cooler in the field, before being transferred (at the close of the day’s fieldwork) to local refrigerated storage. Graders cleaned their hands between examinations with alcohol-based hand sanitizer. At the end of the survey, all swabs were sent to PNG-IMR for storage at −20°C. Swabs were stored frozen at PNG-IMR until testing in October 2018. DNA was extracted using the Qiagen DNA Mini kit according to the manufacturer’s protocol and eluted into 100 μL 10 mM Tris-Cl 0.5 mM ethylenediaminetetraacetic acid.

Prior to testing with the CT assay, conjunctival specimens were tested for presence of host DNA using quantitative PCR (qPCR) targeting the *Homo sapiens* β-globin gene. Only those with host DNA present were tested for CT DNA. To detect CT DNA, a published primer–probe set targeting the CT plasmid was used (forward: 5′-AACCAAGGTCGATGTGATAG-3′, reverse: 5′-TCAGATAATTGGCGATTCTT-3′, probe: 5′-[FAM]-CGAACTCATCGGCGATAAGG-[BHQ1]-3′) [23]. qPCR reactions were made up of 5 μL conjunctival specimen DNA, 7.5 μL of Qiagen QuantiFast Master Mix, forward and reverse primers at 0.25 μM, probe at 0.1 μM, and magnesium chloride at 3 mM and made up to 15 μL with water. A single well was run per conjunctival specimen. The following thermocycling parameters were run on a Bio-Rad CFX96 thermocycler: 3 minutes at 95°C followed by 45 cycles of 5 seconds at 95°C and 1 minute at 55°C. A dilution series of known-concentration cultured C-serovar CT DNA was run on each plate. Baseline fluorescence and the quantitation threshold for the qPCR were set automatically using CFX Manager software. Conjunctival specimens were excluded if the host β-globin target was not amplified within 40 cycles and qPCR positive if the CT plasmid target was amplified within 40 cycles. Linear regression was used to extrapolate CT load from cycle threshold results.

The CT qPCR assay was evaluated by repeat testing of a 10-fold dilution series of C-serovar CT elementary bodies ranging from 14 000 to 0.014 plasmid copies/μL. An external quality assessment panel from Quality Control for Molecular Diagnostics (QCMD; panel reference CTDNA18) was also tested to ensure the assay was performing accurately. The qPCR load estimate of both the dilution series and the QCMD panel were compared to the load determined by droplet digital PCR (ddPCR) carried out at LSHTM. The method for ddPCR testing is described elsewhere [24].

### Anti-Pgp3 Antibodies

Dried blood spots were collected as described previously [12]. Specimens were refrigerated within 1 day, frozen within 1 week, and then shipped at ambient temperatures to the PNG-IMR laboratory. DBSs were then stored at −20°C until shipping at ambient temperatures to CDC for testing. At CDC, they were further stored at −20°C until testing in March–June 2018. Anti-Pgp3 antibodies were measured using a previously described multiplex bead assay (MBA) [25]. One minor modification was made to the published protocol, which was that the immunoglobulin G4 secondary antibody concentration was slightly lower (20 ng/well). The median fluorescence intensity (MFI) was recorded for each sample, and the background from the blank well (elution buffer alone) was subtracted (MFI-bg). The threshold for seropositivity on the MBA was established as an MFI-bg of 1647 using receiver operating characteristic curve analysis on a panel of children from the United States (n = 74) and PCR-positive samples from Tanzania (n = 101).

Seroconversion rates (SCRs) were estimated using reversible catalytic models. We used a Markov chain Monte Carlo simulation approach as described by Pinsent et al [26] to estimate the SCR parameter in the model. We assumed a constant SCR across the ages of 1–9 years. Due to the low number of seropositive individuals, we felt there were insufficient data to estimate seroreversion rate in this population, therefore fixed the seroreversion rate at 0. This corresponded to scenario 1 version 2 in the paper by Pinsent et al [26].

## RESULTS

### PCR Assay Validation

**Figure 2.**
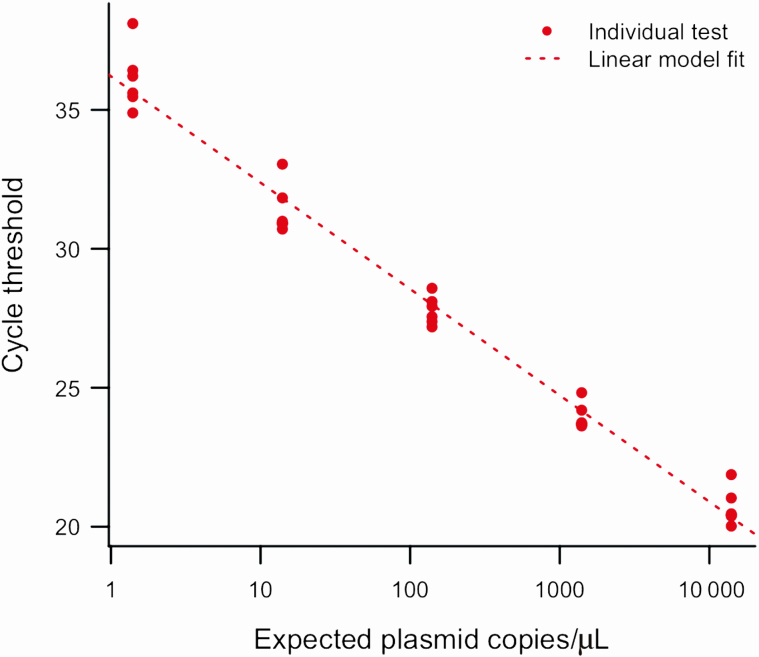
Cycle threshold values from repeat testing of a dilution series of cultured serovar-C *Chlamydia trachomatis* elementary bodies. The coefficient of determination for the linear model is 0.98, and the mean coefficient of variance across the series is 30%. At concentrations <1.4 plasmid copies/μL, *C. trachomatis* DNA was not reproducibly detected.

The lowest CT concentration successfully amplified in 100% of repeat qPCR tests (n = 5) was 1.4 plasmid copies/μL. The mean coefficient of variance across the repeat-tested dilution series was 30%, and the coefficient of determination (*R*^2^) for a linear regression model fitted to the cycle threshold values was 0.98. The cycle threshold values from ocular serovar material repeat tested on validation and clinical test plates are displayed in Figure 2. During testing of the QCMD panel, 8 of 8 positives and 2 of 2 negatives were correctly identified.

### Ocular CT Infection

Across the 3 EUs, 3181 children aged 1–9 years were examined. Four hundred five had TF in 1 or both eyes, of whom 384 had TF in the right eye. Swabs linked to TF case participant information were available for processing from 349 (86%) individuals with TF in either or both eyes. All of these swabs contained PCR-detectable human DNA. Eight (2%) of these had detectable CT plasmid DNA. The median load of infection across the 7 infections detected was 334 plasmid copies/μL (range, 0.4–3628 plasmid copies/μL). The prevalence of infection in children with TF in the 3 EUs ranged from 0 to 4% (Table 1).

**Table 1.** Infection and Serological Findings From Children Aged 1–9 Years Surveyed for Trachoma in Papua New Guinea, October–December 2015

Evaluation Unit	No. Examined^a^	TF, No. (Adjusted Prevalence)^a^	CT Infection Testing			Anti-Pgp3 Antibody Testing		
			Swabs Tested, No.	CT Positive, No.	% (95% CI)	DBSs, No.	Seropositive, No.	% (95% CI)
Western	790	101 (11.2)	93	0	0 (0–4)	728	31	4 (3–6)
Southern Highlands (East)	1391	176 (12.2)	129	5	4 (1–8)	849	55	6 (5–8)
West New Britain	1000	128 (11.4)	127	3	2 (0–6)	995	19	2 (1–3)
Total	3181	405	349	8	2 (1–4)	2572	105	4 (3–5)

Abbreviations: CI, confidence interval; CT, *Chlamydia trachomatis*; DBS, dried blood spot; TF, trachomatous inflammation–follicular in at least 1 eye.

^a^Adjusted for age in 1-year age bands using local census data. Demographic and clinical data published elsewhere [6].

### Anti-Pgp3 Antibodies

**Figure 3.**
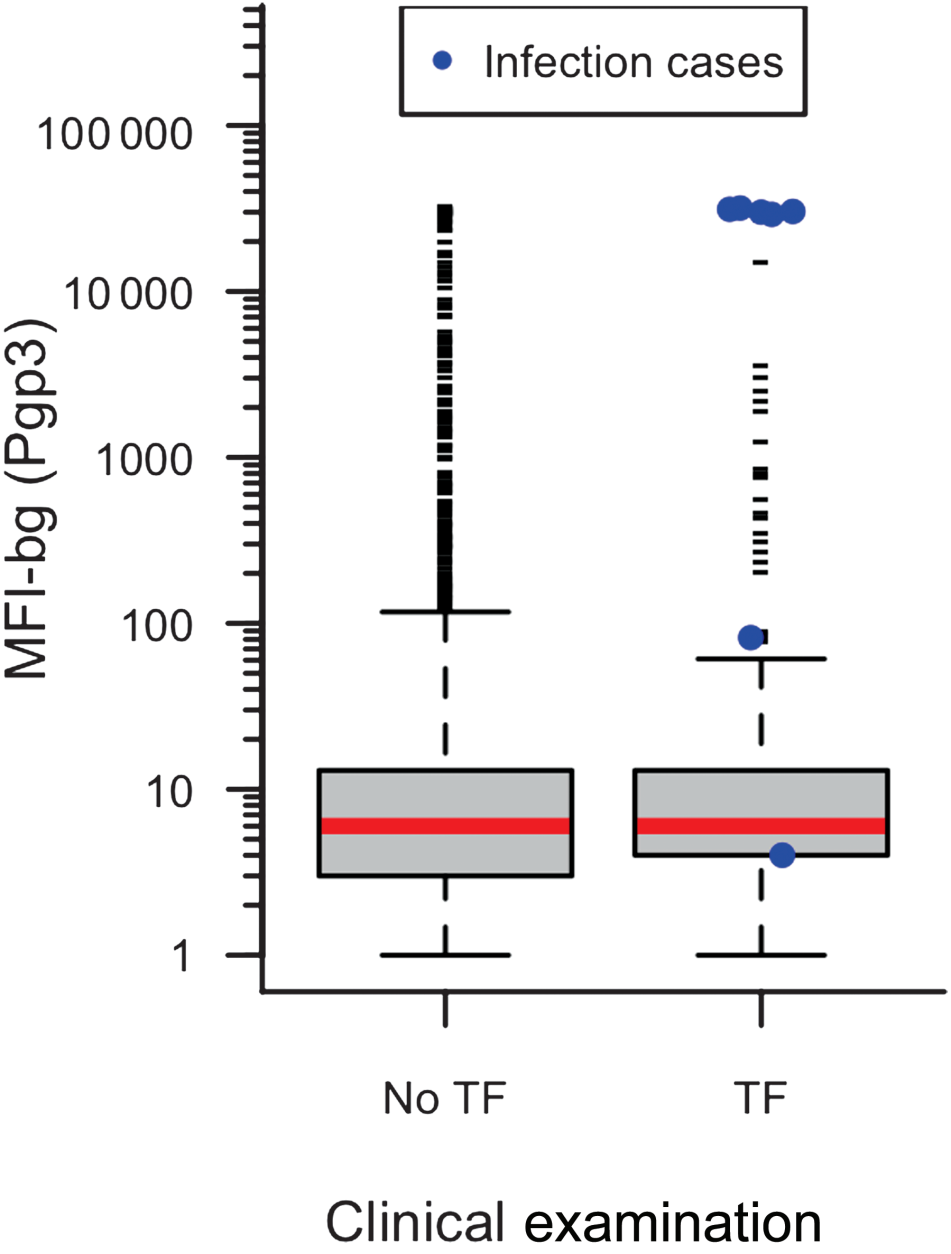
Anti-Pgp3 responses in children from Papua New Guinea aged 1–9 years with and without TF in at least 1 eye, and in children with *Chlamydia trachomatis* infection in the right eye, as detected by quantitative polymerase chain reaction. Specimens were collected in October–December 2015. Boxes represent the interquartile range and whiskers represent 1.5 times the interquartile range; data points outside of that distribution are visualized individually. The threshold for a seropositive result is 1647. Abbreviations: MFI-bg, median fluorescence intensity minus background; TF, trachomatous inflammation–follicular.

DBS were collected from 2572 of the 3181 (81%) examined children. Overall, 105 of 2572 (4%) children were anti-Pgp3 seropositive (Table 1). There was no significant difference in MFI-bg between male and female children (Mann-Whitney *U* test, *P* = .07) or between those with and without TF (Mann-Whitney, *U* test, *P* = .77; Figure 3). Of the 7 individuals who tested positive for infection, 5 were seropositive. Individuals with infection had significantly higher anti-Pgp3 responses than their infection-negative counterparts (Mann-Whitney *U* test, *P* < .0001; Figure 3).

**Figure 4.**
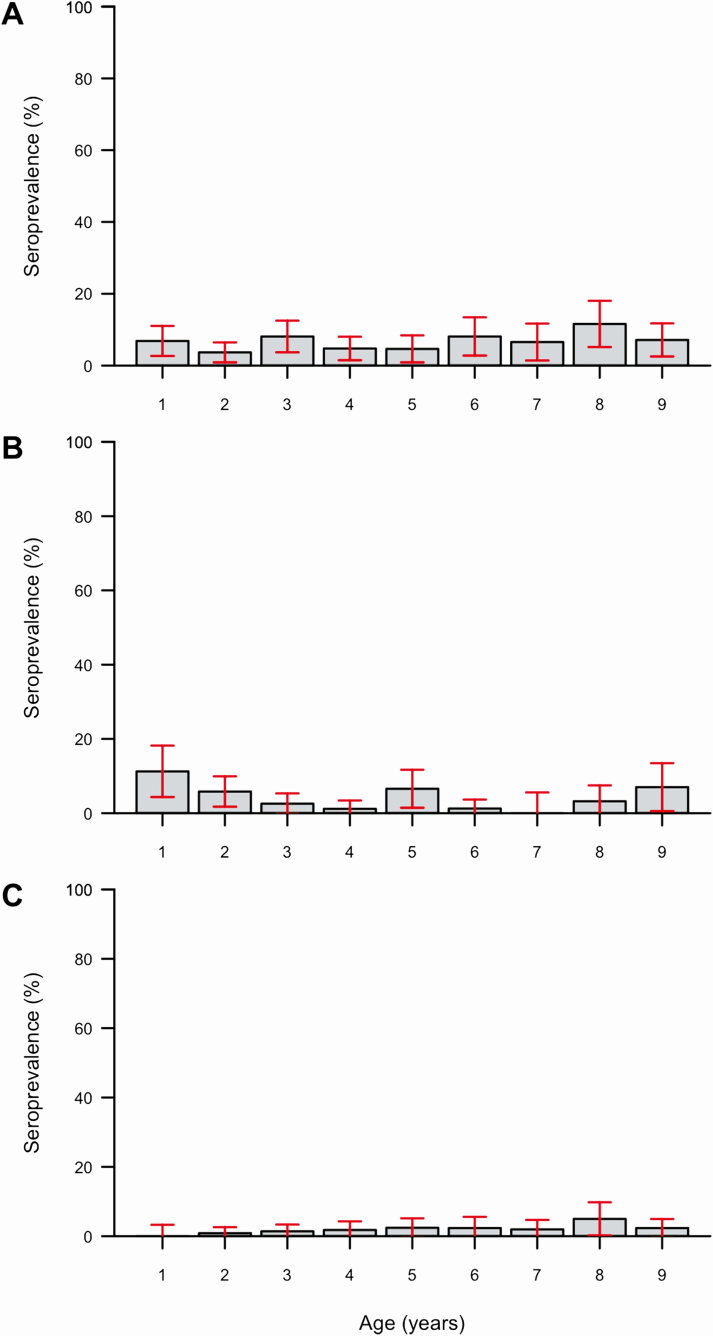
Age-specific anti-Pgp3 seroprevalence in children aged 1–9 years living in Southern Highlands (east) (*A*), Western (*B*), and West New Britain (*C*) provinces of Papua New Guinea, October–December 2015. Bars represent age-specific seroprevalence, and whiskers represent 95% confidence intervals.

Considering data combined from all 3 EUs, there was no statistically significant increase in seroprevalence with age (logistic regression, *P* = .54). Examining the data for each EU separately, there was no statistically significant increase in seroprevalence with age in Southern Highlands (east) or in Western Province (logistic regression, *P* = .19 and *P* = .32, respectively) (Figure 4A and 4B). In West New Britain, there was a significant positive association between seroprevalence and increasing age (logistic regression, *P* = .01), yet the maximum age-specific seroprevalence was 5% (in 8-year-olds). The proportion of children in West New Britain who were seropositive was very low (≤ 5% in all age groups) (Figure 4C). The estimated SCRs in Southern Highlands (east), Western, and West New Britain provinces were, respectively, 0.016 (95% credible interval [CrI], .011–.020), 0.010 (95% CrI, .006–.013), and 0.004 (95% CrI, .003–.006) seroconversion events per individual per year.

## Discussion

According to current WHO definitions based on clinical signs [27], trachoma is a public health problem in all 6 mapped EUs of PNG, and a regimen of 3 rounds of azithromycin is indicated in 4 of the 6 EUs. Here, we undertook further investigations in 3 EUs: Southern Highlands (east) (baseline TF prevalence in 1- to 9-year-olds, 12.2% [95% confidence interval {CI}, 9.6–15.0]), Western Province (11.2% [95% CI, 6.9–17.0]), and West New Britain (11.4% [95% CI, 8.7–13.9]) [6]. We used specimens collected during the baseline mapping fieldwork that generated those prevalence estimates, and demonstrated that in all 3 EUs, the vast majority of children with TF did not have CT infection at the time of the baseline surveys. We also demonstrated that more than 95% of individuals with TF were seronegative for anti-CT antibodies, suggesting that they had had little or no exposure to CT prior to the surveys. In 2 of 3 tested EUs, there was no evidence that seroresponsiveness to Pgp3 accumulated through childhood to the age of 9 years. In West New Britain, a statistically significant increase in age-specific seroprevalence was observed, but the overall seroprevalence was very low, even in 8- and 9-year-olds.

This pattern in PNG closely resembles that seen in the Solomon Islands and Vanuatu, where the prevalence of TF is high in children, but TT is rarely seen in adults [7, 8]. In the Solomon Islands and Vanuatu, the prevalence of ocular CT infection in children with TF was 3.9% and 1.8%, respectively [11, 28], and anti-Pgp3 seroprevalence did not increase with age [12]. By contrast, the prevalence of ocular CT infection on Kiritimati Island in Kiribati is 24% among children; there, anti-Pgp3 seroprevalence among 1- to 9-year-olds exceeds 50% [29]. Considered together, these data suggest that ocular CT infection is currently rare in children in this part of PNG and is not being intensively transmitted, despite a prevalence of the clinical sign (TF) indicative of a need for public health interventions.

There are several possible explanations for the discordance between TF in children and TT in adults in Melanesian populations. The prevalence of trachoma could be increasing after having been relatively low or absent for a long period. This would mean that the nascent infection had not been present for long enough to produce the long-term scarring effects necessary for TT [30]. Although plausible in some respects, this is inconsistent with the relative lack of ocular CT infection and anti-Pgp3 antibodies that we identified here in young children, where the highest levels of infection are typically seen. An alternative explanation may be that trachoma is in decline in this population, resulting in an attenuation of the relationship between CT infection and TF. This is inconsistent with the relative absence of TT in adults, unless the tailing off of the secular decline in TF prevalence is extremely long. In these cross-sectional surveys, it is not possible to draw any conclusions about temporal changes in TF prevalence, but in longitudinal data from studies in sub-Saharan Africa, it is apparent that as trachoma disappears, the decrease in TF prevalence lags behind the decrease in ocular CT infection over years, not decades. We would therefore expect to see many more cases of TT in PNG [14, 31–33] if this were the explanation for the discordance. A third explanation could be that a high prevalence of urogenital CT infection in the population might enable individuals in these populations to better control ocular CT infections. The most likely explanation, however, for the high prevalence of TF in the absence of TT in these populations is that a proportion of TF in Melanesia is follicular inflammation triggered by something other than CT infection, with the “trachomatous” part of the sign’s name being misleading. In an area of Tanzania with low CT infection prevalence, TF was more associated with ocular *Streptococcus pneumoniae* and *Haemophilus influenzae* infection than CT infection [34]. None of these 4 possibilities has yet been empirically proven in Melanesia, nor have similar scenarios been sufficiently extensively investigated in other parts of the world.

There are limitations to this study. First, because the study was time- and resource-limited, conjunctival specimens were collected only from the right eye, and only then in children who had TF. It is therefore not possible to determine (*i*) the absolute community prevalence of ocular CT infection or (*ii*) the association between TF and ocular CT infection. By testing both eyes and more children, we may have learned more about the true burden of infection. One specific potential source of additional infection cases that would not have been identified in this study is the group of children with conjunctival inflammation not meeting the criteria for TF (eg, those with < 5 follicles), as these individuals are more likely to have CT infection than those with no follicles [35]. As WHO’s trachoma recommendations do not currently use ocular CT prevalence to guide interventions, this was considered an acceptable compromise given the time and resource constraints. Investigating the association between TF and CT infection more fully in this setting could have contributed to our understanding of trachoma in the region.

A second limitation of the current study was that serological data were missing from 19% of children and infection data were missing from 12% of children with TF. No data were collected on reasons for refusal of testing; therefore, we cannot make any assumptions about the relative importance of the missing data. Third, seroprevalence estimates in each EU are so low that the specificity of the test may have a disproportionally large effect on study outcomes. For example, West New Britain had the lowest overall seroprevalence of the 3 EUs at only 2%, which is within the reported range of specificity of the Pgp3 MBA [25, 36]; the slight increase in seropositivity with age here should be interpreted with caution. The absolute seroprevalence suggests that a low proportion (5%) of 1- to 9-year-olds have been exposed to CT. Even this low level is probably an overestimate of previous ocular CT exposure. Finally, Pgp3 is not a biovar-specific antigen and the prevalence of urogenital CT in women in PNG is high [37]. Recent studies have reported that ≥ 20% of women tested in PNG antenatal clinics are CT positive [37, 38], so at least some of the seroreactivity in our participants is likely to have been caused by acquisition of infection during parturition [39], or by chance inoculation of urogenital CT into children’s eyes due to poor parental hand hygiene, causing paratrachoma. This reinforces our conclusion that trachoma is unlikely to be a public health problem in this population.

A growing body of work suggests that TF has low specificity for infection after MDA and in low-prevalence environments. As those environments become increasingly common, defining active trachoma purely in terms of the presence of follicular conjunctival inflammation will invite challenge. In light of the data presented here and the accumulated evidence from elsewhere in Melanesia, it is clear that we do not yet fully understand the burden of nontrachomatous follicular inflammation and the implications it has for the use of TF < 5% to define “elimination of trachoma as a public health problem” in some settings. Criteria for initiating antibiotic MDA to eliminate trachoma should be reviewed, and use of non-TF markers of CT transmission should be considered for routine trachoma mapping exercises.

## Notes

***Acknowledgments.*** The authors thank Harry Pickering (London School of Hygiene and Tropical Medicine [LSHTM]) for providing cultured *Chlamydia trachomatis* material to evaluate the diagnostic assay used in this study; Barne Willie and Ben Toto (Sexual and Reproductive Health Unit, Papua New Guinea Institute of Medical Research [PNG-IMR]) for assistance with the Chlamydia trachomatis quantitative polymerase chain reaction assay; and Amy Pinsent and Nuno Sepúlveda (LSHTM) for support in the seroconversion rate modeling.

***Financial support.*** Fieldwork for collection of specimens used in this study was funded by the Global Trachoma Mapping Project (GTMP), supported by a grant from the United Kingdom’s Department for International Development (ARIES: 203145) to Sightsavers, which led a consortium of nongovernmental organizations and academic institutions to support health ministries to complete baseline trachoma mapping worldwide. The GTMP was also funded by the United States Agency for International Development, through the ENVISION project implemented by RTI International (grant number AID-OAA-A-11-00048), and the End Neglected Tropical Diseases in Asia project implemented by FHI360 (OAA-A-10-00051). A. W. S. was a Wellcome Trust Intermediate Clinical Fellow (number 098521) at LSHTM. R. B. was funded by the Fred Hollows Foundation (number 1954-0). Funding for processing of specimens was provided by the International Trachoma Initiative in awards to the PNG-IMR.

***Potential conflicts of interest.*** The authors: No reported conflicts of interest. All authors have submitted the ICMJE Form for Disclosure of Potential Conflicts of Interest.
